# From Early Domesticated Rice of the Middle Yangtze Basin to Millet, Rice and Wheat Agriculture: Archaeobotanical Macro-Remains from Baligang, Nanyang Basin, Central China (6700–500 BC)

**DOI:** 10.1371/journal.pone.0139885

**Published:** 2015-10-13

**Authors:** Zhenhua Deng, Ling Qin, Yu Gao, Alison Ruth Weisskopf, Chi Zhang, Dorian Q. Fuller

**Affiliations:** 1 School of Archaeology and Museology, Peking University, Beijing, China; 2 Institute of Archaeology, University College London, London, United Kingdom; National Institute of Plant Genome Research (NIPGR), INDIA

## Abstract

Baligang is a Neolithic site on a northern tributary of the middle Yangtze and provides a long archaeobotanical sequence from the Seventh Millennium BC upto the First Millennium BC. It provides evidence for developments in rice and millet agriculture influenced by shifting cultural affiliation with the north (Yangshao and Longshan) and south (Qujialing and Shijiahe) between 4300 and 1800 BC. This paper reports on plant macro-remains (seeds), from systematic flotation of 123 samples (1700 litres), producing more than 10,000 identifiable remains. The earliest Pre-Yangshao occupation of the sites provide evidence for cultivation of rice (*Oryza sativa*) between 6300–6700 BC. This rice appears already domesticated in on the basis of a dominance of non-shattering spikelet bases. However, in terms of grain size changes has not yet finished, as grains are still thinner than more recent domesaticated rice and are closer in grain shape to wild rices. This early rice was cultivated alongside collection of wild staple foods, especially acorns (*Quercus/Lithicarpus* sensu lato). In later periods the sites has evidence for mixed farming of both rice and millets (*Setaria italica* and *Panicum miliaceum*). Soybean appears on the site in the Shijiahe period (ca.2500 BC) and wheat (*Triticum* cf. *aestivum*) in the Late Longshan levels (2200–1800 BC). Weed flora suggests an intensification of rice agriculture over time with increasing evidence of wetland weeds. We interpret these data as indicating early opportunistic cultivation of alluvial floodplains and some rainfed rice, developing into more systematic and probably irrigated cultivation starting in the Yangshao period, which intensified in the Qujialing and Shijiahe period, before a shift back to an emphasis on millets with the Late Longshan cultural influence from the north.

## Introduction

It is well established that early farming in China can be divided into a group of northern traditions based on the cultivation of millets (*Setaria italica* and *Panicum miliaceum*) and southern tradition in the Middle and Lower Yangtze basin focused on rice (*Oryza sativa*) cultivation [1]. What is less clear is the interrelationships between these two archaeological traditions. Some have argued for a single origin, with either rice agriculture spreading north and encouraging millet domestication [2,3], or early millet farming in the north spreading south and kick-starting millet cultivation [4,5]. Others have argued for more than independent center of millet domestication across north China, unconnected to Middle and Lower Yangtze basin rice domestication episodes [6–8]. It is nevertheless clear that rice spread north into the millet focused Yellow River basin, perhaps by 4000 BC [9–11]], and that foxtail millet had been adopted into the rice-growing middle Yangtze before 4000 BC [12, 13]. What has been scarce are sites that provide a long chronological sequence that allow for documenting the ebb and flow of rice and millet agriculture between the Yellow and the Yangtze basins. The present article report data from the Baligang excavation project of Peking University which provides a sequence of archaeobotanical data between the 7^th^ and 2^nd^ millennia BC for the Nanyang Basin, an area on a northern tributary of the middle Yangtze, near a route of communication to the Yellow river.

**Fig 1:**
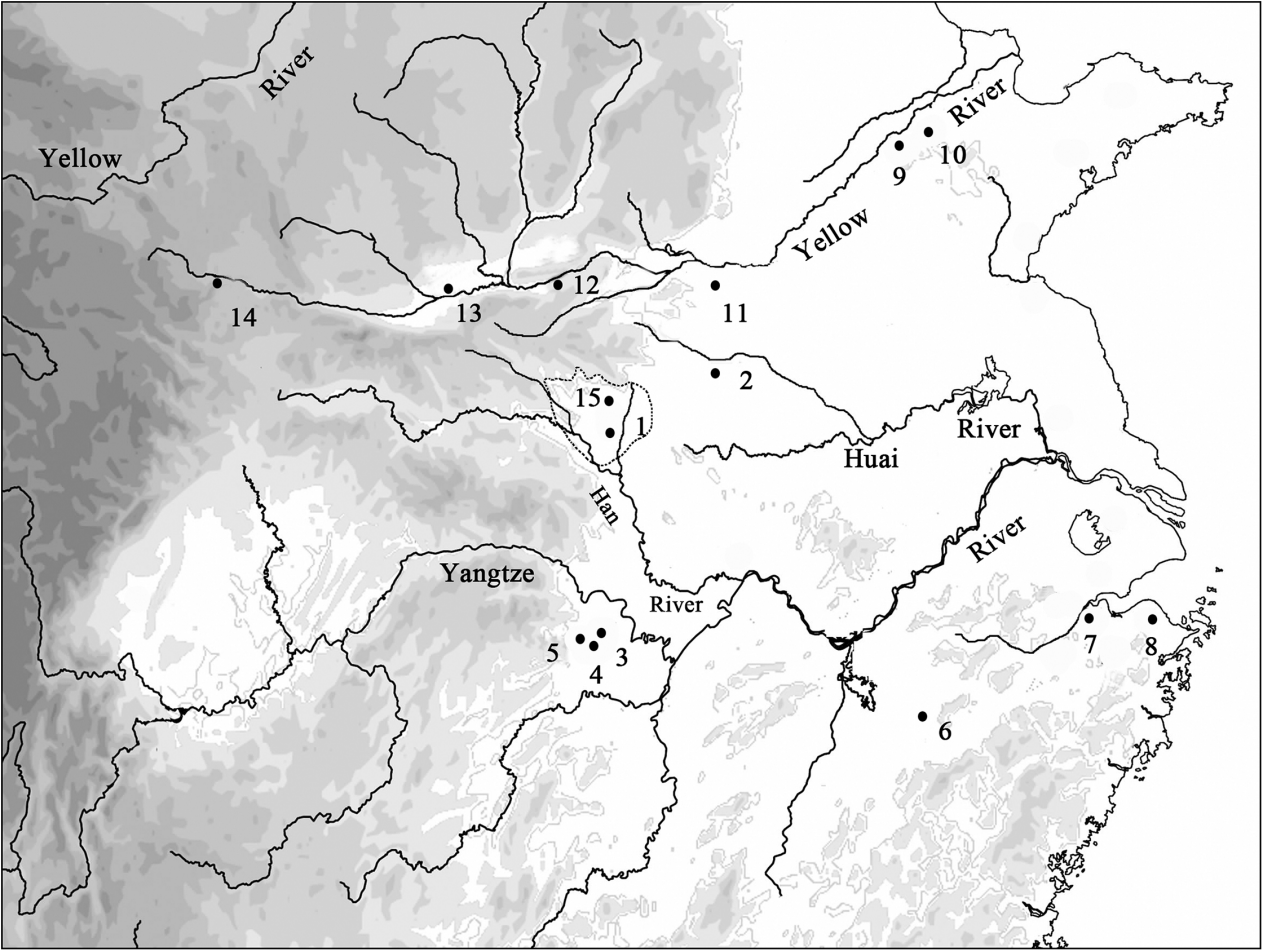
Map locating the Nanyang Basin, Baligang and other sites discussed in this article: *1*. *Baligang 2*. *Jiahu 3*. *Bashidang 4*. *Pengtoushan 5*. *Chengtoushan 6*. *Xianrendong*, *7*. *Kuahuqiao 8*. *Tianluoshan 9*. *Yuezhuang 10*. *Xihe 11*.*Peiligang 12*.*Nanjiaokou*, *13*. *Xinglefang 14*. *Xishanping 15*.*Huitupo*

The Nanyang basin, situated in the southwest part of Henan Province, China, is surrounded by mountains on three sides, with the Qinling Mountain to the west, Funiu Mountain to the north, and Tongbai Mountain to the east. Originating from the Funiu Mountain, the main branches of Tangbai River flow over most parts of the basin from north to south, and finally fall into the Han River, which is an important tributary of the Yangtze River (Fig 1). Geographically, Nanyang basin falls on the traditional dividing line between north and south China, the Qingling-Huaihe line, with Qinling Mountain to the west and Huai River originating from the east edge of the basin. Meanwhile, it also lies between the basins and plateaus of the middle elevations of southwest China and the broad plains and lower elevation of eastern China. Benefiting from this transitional location, Nanyang Basin enjoys a quite good climate with adequate rainfalls (700–1200 mm average per annum) and fairly light winters (0.5–2.4°C average in January). On the whole, the special natural and cultural location eventually leads to the unique status of the Nanyang basin in terms of cultural development, as falling at the cultural transition between the Yellow River cultures and the Yangtze River cultures. As an agricultural region it is suitable for both dry-land agriculture (millets, wheat) and paddy-field agriculture.

In general, North China is accepted as a dry-land farming area based on foxtail millet (*Setaria italica*) and broomcorn millet (*Panicum milliaceum*) in the Neolithic period. While South China has continuously been a rice farming area at least after the middle Neolithic age, since ca. 4500 BC [1, 14]. However, plant evidence from more and more sites reveal that mixed farming existed in the Neolithic age of north and south China [11, 13, 15–18]. This raises questions about the diffusion process of these two kinds of crops into each other’s territory and the cultural motivations behind crop diffusion. In this case, the transitional area between north and south China, such as the Nanyang basin, becomes a critical region for us to examine the formation of this kind of mixed farming, the changing proportions of different crops, as well as the impacts of natural and cultural factors.

**Fig 2:**
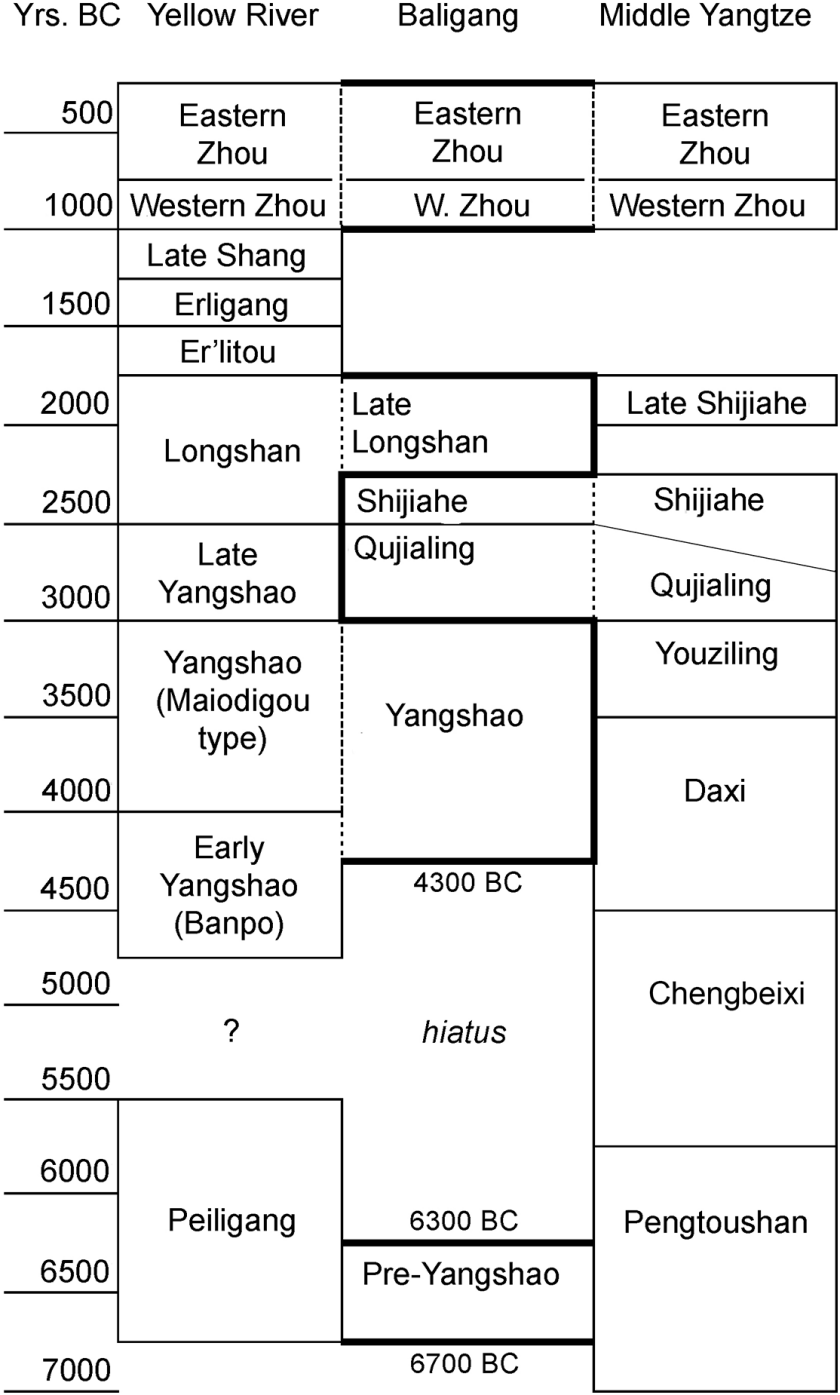
Chronology of Baligang in comparison to Yellow River and Yangtze Chronologies.

Baligang is a settlement site located in the middle of Nanyang basin, and its various phases of occupations have cultural affiliations that vary between north and south (Fig 2). The earliest remains found here are 11 ash pits belonging to Pre-Yangshao period (ca.6700–6300BC) [19], which are similar in age to the Jiahu site located in the east on the Huai River [20]. Details of cultural affiliation of this period at Baligang are poorly understood due to a small assemblage and only distant comparisons. It is referres as “Pre-Yangshao” [19, 20] because it is stratigraphically below the Yangshao occupation at the site. After a hiatus, it was used as a sedentary settlement in the Yangshao period (ca.4300–3000BC), with its cultural material related to the Yellow River region. Cultural material from this period has been studied over ten seasons of excavation. These excavations revealed house foundations, cemetery, and storage pits, which backfilled with ancient ashy midden after being abandoned. Subsequent to that period are archaeological remains indicating affiliation with the Yangtze cultural sequence, including Qujialing (ca.3000–2500BC), and Shijiahe (ca.2500–2300BC) periods, represented by houses, pits (probable storage pits) and urn burials. Above these are levels of pits and burials of the Late Longshan culture (ca.2300–1800BC) and pits of the Bronze Age (mainly Western and Eastern Zhou, 1046–256 BC), which once again point to northern cultural influences [21–23].

## Materials and Methods

Flotation samples for archaeobotanical analysis were collected from pits and cultural layers during the excavation seasons in 2004 and 2007. In all, more than 1700 liters soil of 123 samples were floated by flotation tank or bucket at the site (Table 1). Plant remains were collected by sieves with 0.3mm mesh. After drying at the site, samples from excavation in 2004 were sent to the archaeobotany laboratory of Institute of Archaeology UCL, and samples from 2007 to the archaeobotany laboratory of School of Archaeology and Museology PKU for further identification and analysis. In addition, sediments from many of the same contexts were processed for phytolith assemblages [24, 25].

**Table 1:** Periods and contexts of archaeobotanical samples from Baligang site.

Period	No. of samples	Pit fills	Cultural layers
Pre-Yangshao (ca. 6700—6300BC)	11	11	
Yangshao (ca.4300—3000BC)	14	14	
Qujialing (ca.3000—2500BC)	27	26	1
Shijiahe (ca.2500—2300BC)	19	19	
L.Longshan (ca.2300—1800BC)	44	42	2
Western Zhou (ca.1046 -771BC)	1		1
Eastern Zhou (770—256BC)	7	2	5
Total	123	114	9

Before being sorted under a binocular stereomicroscope, every sample was weighed and measured, and then sifted in sample sieves. In most samples charcoals and fragments smaller than 0.3mm were not sorted, as a sorting test showed no seeds and fruits are to be found in these tiny remains. Excluding this < 0.3mm fraction, all samples were completely sorted. Seeds, fruits and other parts of plants were separated from charcoals and identified with reference to modern comparative collections and Chinese seed atlases [26]. All finds are sorted by taxon in plastic tubs labelled with taxon and context number, and stored in the Archaeobotany laboratory at Peking University (Bejing, 100871, China), apart from a few specimens that were attached to metal stud for scanning electron microscopy, which are boxed separately.

Selected crop and seed specimens from throughout the seqeucne were selected for direct radiocarbon dating by accelerator mass spectrometry (AMS). These specimens were single grains of rice or wheat or other plant remains. In the case of millet (*Setaria italica*) three grains were combined from the same flotation sample in order to assure enough carbon after pretreatment. These samples were analyzed at the Peking University AMS radiocarbon facility (BA), the National Key Laboratory for Radiocarbon dating in China.

## Results

Over the course of excavations, the chronology was initially based on material cultural affiliation. Fifteen dates were carried out on wood charcoal in support of this. In addition 18 radiocarbon dates were direct AMS dates on identified seed remains recovered from the archaeobotanical samples described in this paper. These short-lived samples provide precise dates and support the cultural sequence developed by the excavators (Fig 2), The direct AMS-radiocarbon dates on short-lived material, mainly rice grains, summarized in Table 2.

**Table 2:** Summary of AMS radiocarbon dates of the Baligang site on short-lived samples.

Lab Code	Sample type	Context No.	Cultural period	Uncalibrated C-14 date BP (5568)	Calibrated age range (95.4%)
BA111568	Rice grain	H1981②	Pre-Yangshao	7680±30	6600BC (95.4%) 6460BC
BA111569	Rice grain	H1985	Pre-Yangshao	7690±25	6600BC (95.4%) 6460BC
BA111571	Rice grain	H1992	Pre-Yangshao	7710±25	6600BC (95.4%) 6470BC
BA08119	Fruit husk	H2000	Pre-Yangshao	7445±55	6430BC (95.4%) 6220BC
BA111570	Unidentified plant fragment	H1991	Yangshao	5320±25	4240BC (95.4%) 4050BC
BA081045	Unidentifie endocarp fragment	H1966	Yangshao	5355±40	4330BC(13.4%)4280BC 4270BC(82.0%)4050BC
BA111566	Rice grain	H1966③	Yangshao	5350±25	4320BC (7.1%) 4290BC 4270BC (88.3%) 4050BC
BA081046	Rice grain	H1958	Yangshao	5085±35	3970BC(95.4%)3790BC
BA081047	Rice grain	H1957	Yangshao	5035±35	3950BC(95.4%)3710BC
BA081048	Rice grain	H1780	Qujialing	4195±40	2900BC(25.6%)2830BC 2820BC(68.1%)2660BC 2650BC (1.7%) 2630BC
BA081049	Rice grain	H1929	Qujialing	4130±40	2880BC(95.4%)2580BC
BA081050	Rice grain	H1949①	Qujialing	4155±35	2880BC(95.4%)2620BC
BA081051	Rice grain	H1876②	Qujialing	3835±35	2460BC(94.4%)2190BC 2160BC (1.0%) 2150BC
BA081052	Rice grain	H114	Shijiahe	3955±35	2580BC(95.4%)2340BC
BA120105	Rice grain	H1769	Shijiahe	3895±35	2480BC (95.4%) 2280BC
BA081053	Rice grain	H1880	Bronze Age	2560±35	810BC (52.3%) 730BC 690BC (15.3%) 660BC 650BC (27.8%) 540BC
BA081054	Foxtail millet	H1880	Bronze Age	2935±35	1270BC(95.4%)1020BC
BA081055	Wheat grain	H1880	Bronze Age	2500±35	790BC (94.2%) 500BC 440BC (1.2%) 420BC

### Archaeobotanical assemblage

**Fig 3:**
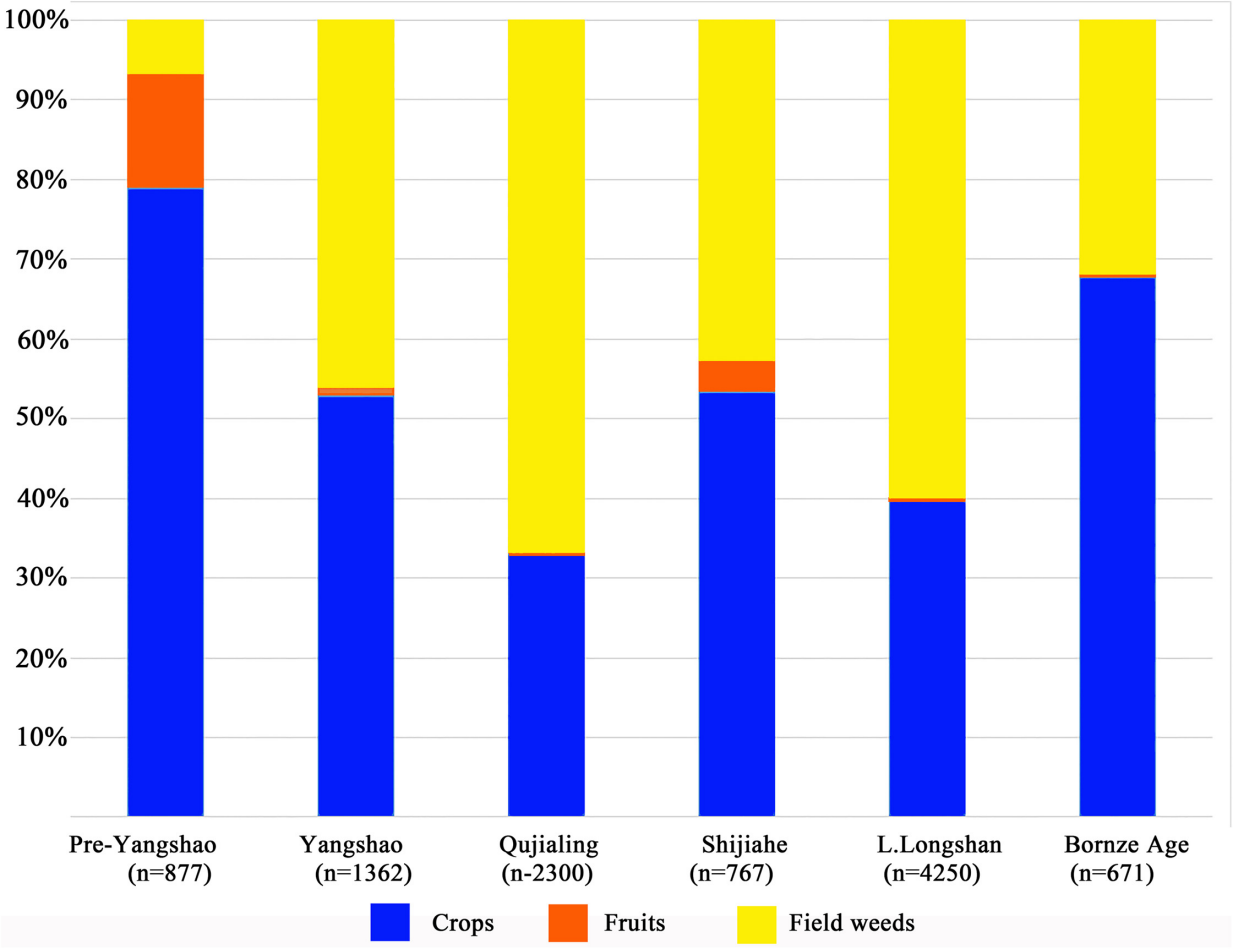
Proportions of main plant categories in assemblages of each period of the Baligang site.

**Fig 4:**
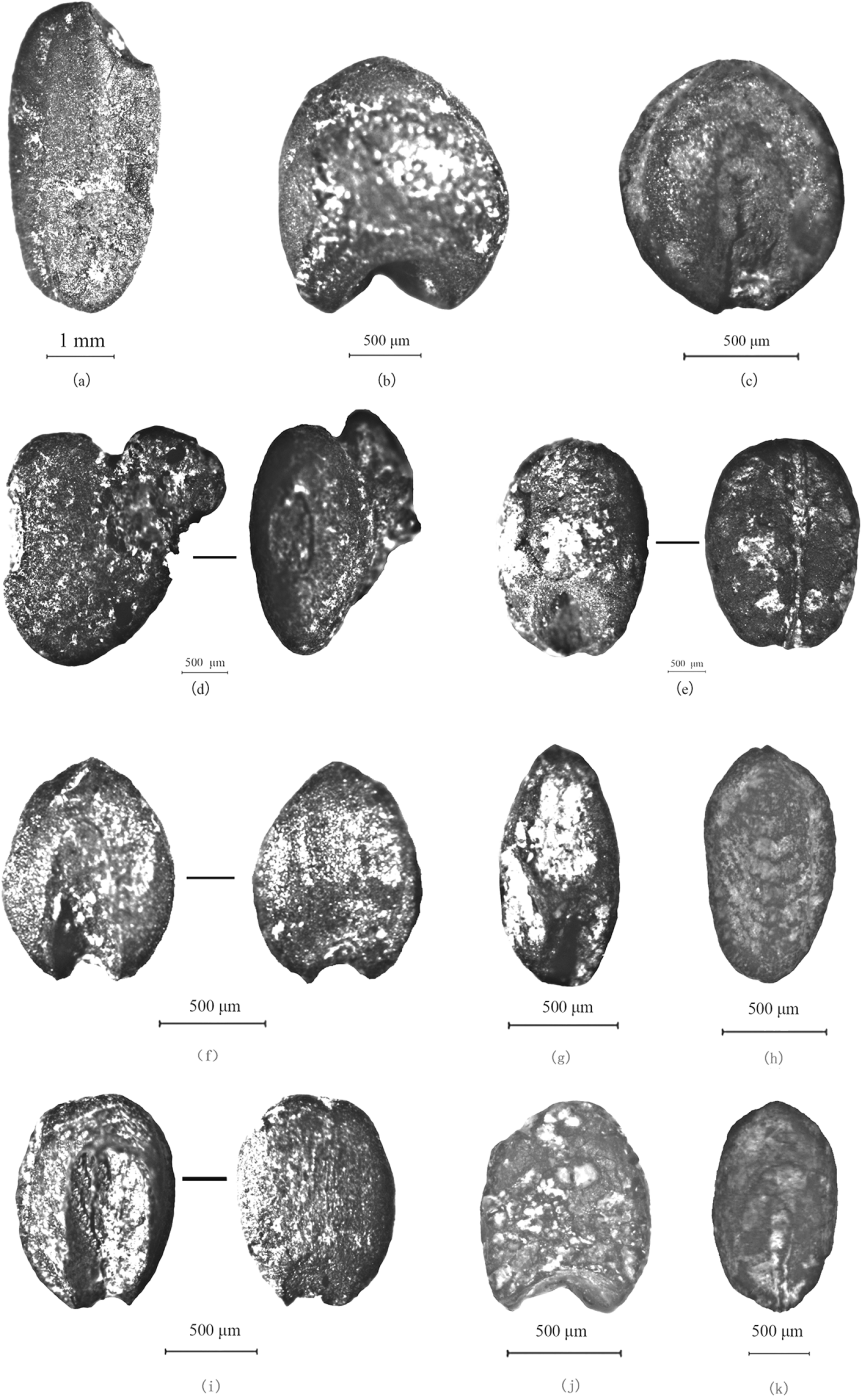
Photomicrographs of representative specimens of crop types and grassy weeds from Baligang site: a. *Oryza sativa* grain (H1958) b. *Panicum milliaceum* grain (H1211) c. *Setaria italica* grain (H1211) d. *Glycine* cf. *max* seed (H1211) e. *Triticum* cf. *aestivum* grain (H1880) f. *Setaria viridis* (H1880) g. *Digitaria sp*. (H1880) h. *Eleusine indica* (H1982) i. *Echinochloa sp*. (H1880) j. *Panicum sp*. (H2000) k. cf. *Bromus sp*. (H1925).

**Fig 5:**
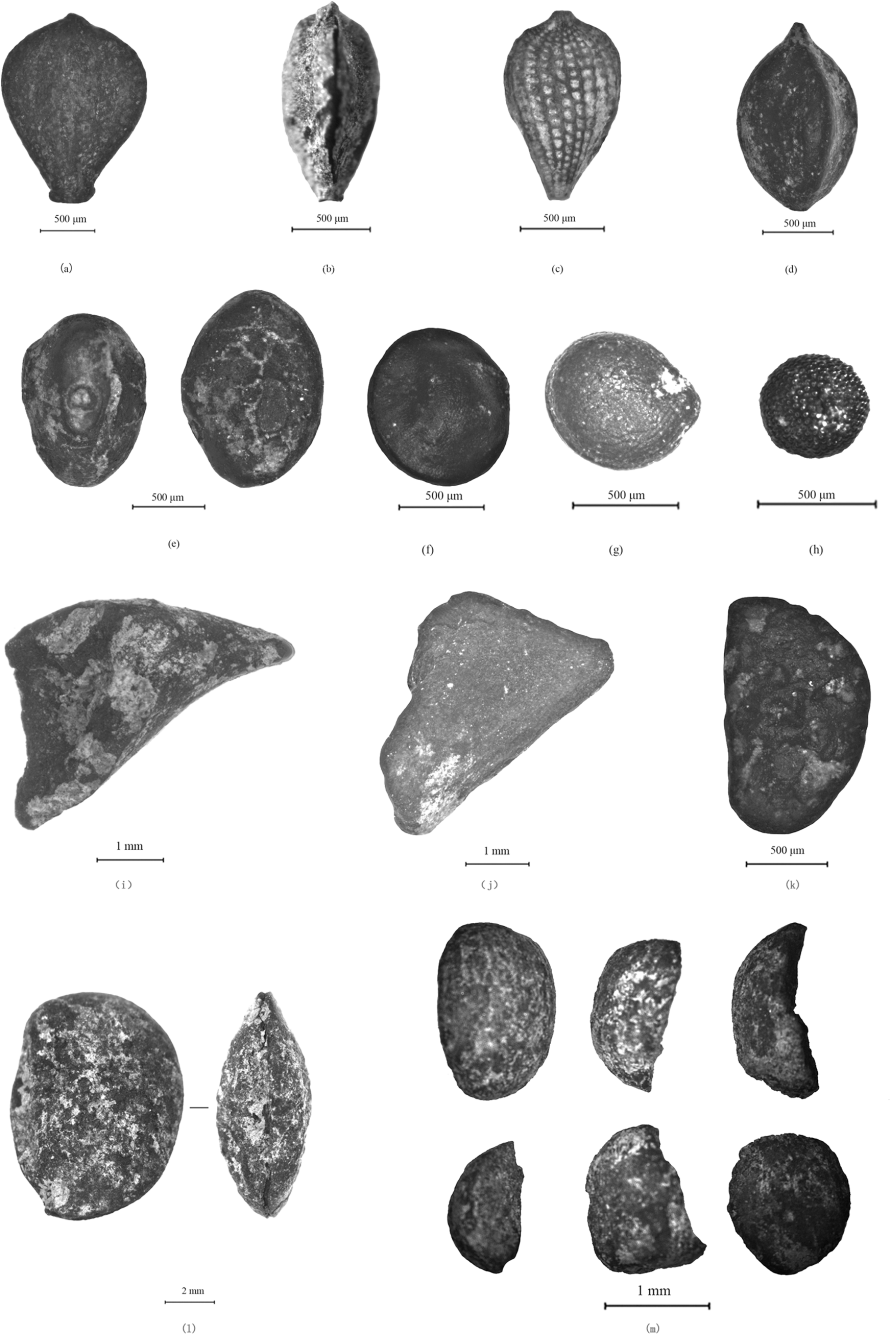
Photomicrographs of other weeds, fruits and nut types from Baligang site: a. *Scirpus sp*. (H1949) b. *Cyperus sp*. (H1949) c. *Fimbristylis sp*. d. *Rumex sp*. (H1822) e. *Legume* (H1822) f. Amaranthaceae (H1880) g. *Chenopodium sp*. (H1818) h. *Brassica sp*. (H2000) i. *Trapa sp*. ‘horn’ (H1211) j. *Trapa sp*. nutmeat (H1211) k. *Rubus sp*. (H1981) l. *Diospyros sp*. (H1822) m. fragments of acorns (H2000).

A total of 53 species and more than 10082 plant remains were identified from 123 samples (full dataset is S1 Table). All of them can be classified into four categories, comprising of 5 species of crops, 6 species of fruits or nuts, edible aquatic plants, and 42 species of possible field weeds. Crops are the most important part in the assemblage of plant remains, taking up more than 50% of all remains in most periods. Except for Pre-Yangshao period, archaeobotanical evidence from the later periods tends to demonstrate a crop-based subsistence strategy, while wild fruits just make up a very small proportion in all the edible plants from the Baligang site (Fig 3). Representatives of the main crops and major wild taxa are illustrated in Figs 4 and 5. Wild taxa occupying more than 40% in the plant assemblages of most periods, are likely field weeds.

### Crops

**Fig 6:**
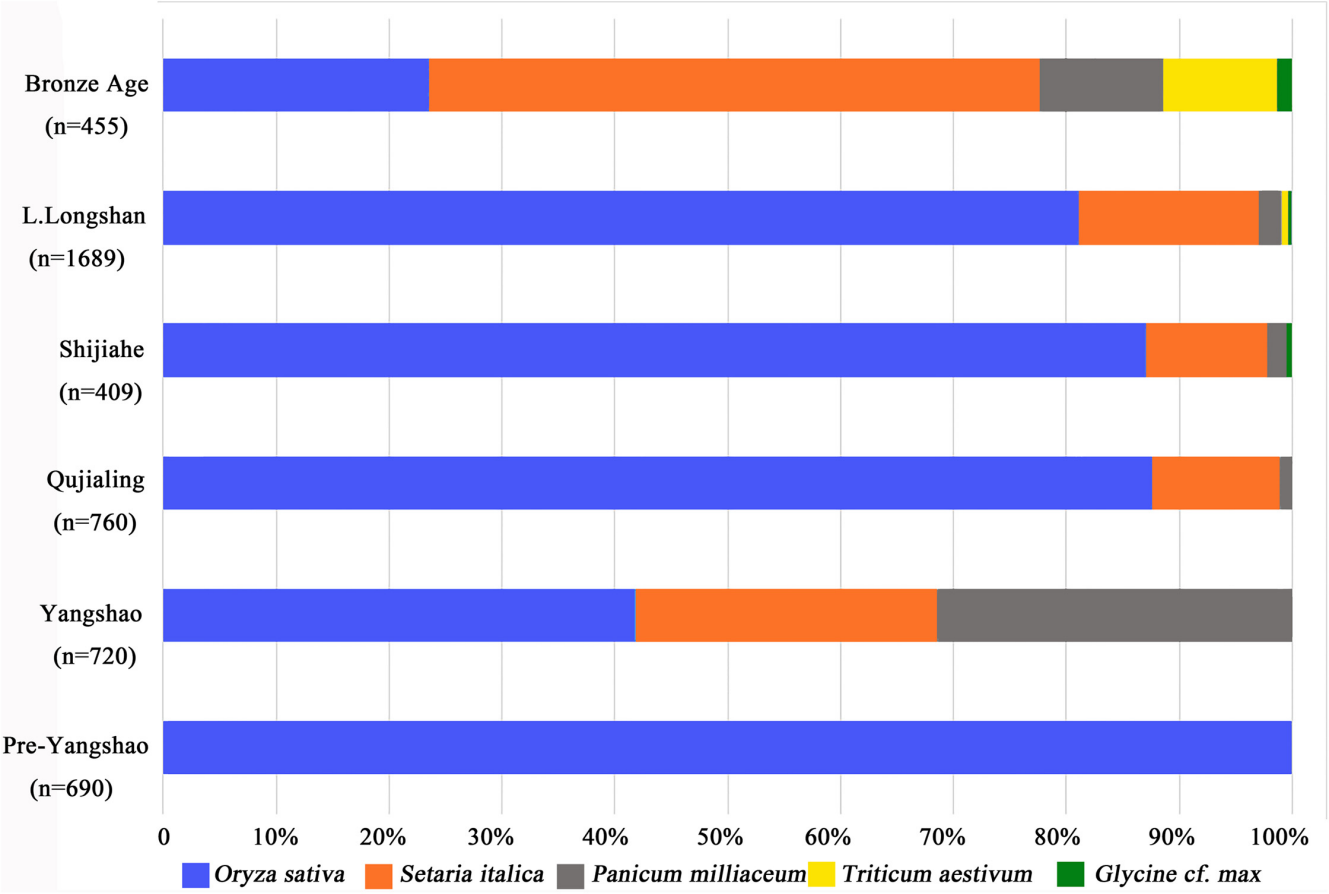
Proportion of different crops in all periods of Baligang site.

**Fig 7:**
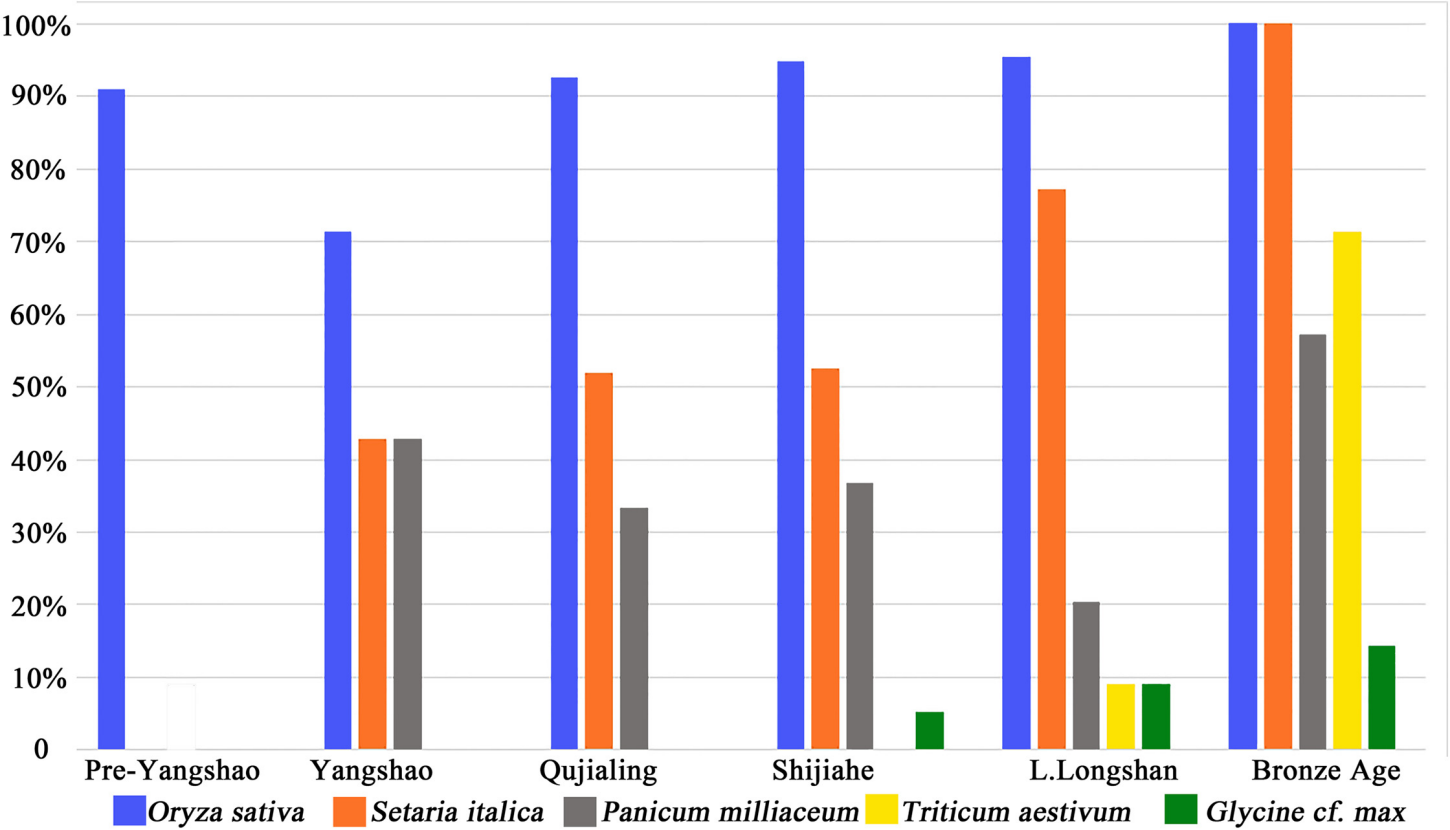
Ubiquity of different crops in all periods of Baligang site.

Patterns in the relative frequency (Fig 6) and ubiquity (Fig 7) indicate the basis of the agricultural economy. Rice (*Oryza sativa*), foxtail millet (*Setaria italica*), broomcorn millet (*Panicum Milliaceum*) and wheat (*Triticum* cf. *aestivum*) are the four main types of domesticated crops found at Baligang site (Fig 4). Soybean (*Glycine* cf. *max*), thought to be domesticated in the Yellow River region over the course the Yangshao and Longshan periods [27, 28], appears in the Shijihe period and may be a minor crop. A small amount of small *Glycine* cf. *soja* seeds in the Pre-Yangshao period likely represent wild collecting.

The most important crop was rice in all periods of Neolithic Age, while foxtail millet seemed to be the second in importance in the Neolithic but became the dominant staple in the Bronze Age. During the Yangshao period, foxtail and broomcorn millet together outnumbered rice, and in this way the Yangshao data [9–11, 18] indicate northern millet-growing traditions than to contemporary Daxi period Yangtze [13, 29]. Broomcorrn millet made a minor comeback in the Bronze Age. Wheat was first introduced into this site in the Late Longshan period, and was just a tiny part in the crop assemblage (0.53%). Even in the Bronze Age, the proportion of wheat was still quite low (10.11%), although it had slightly increased compared to the Neolithic Age. Besides, soybean (*Glycine* cf. *max*) could be another possible crop especially after the Shijiahe period, although its frequency and ubiquity are both very low (Figs 6 and 7).

**Fig 8:**
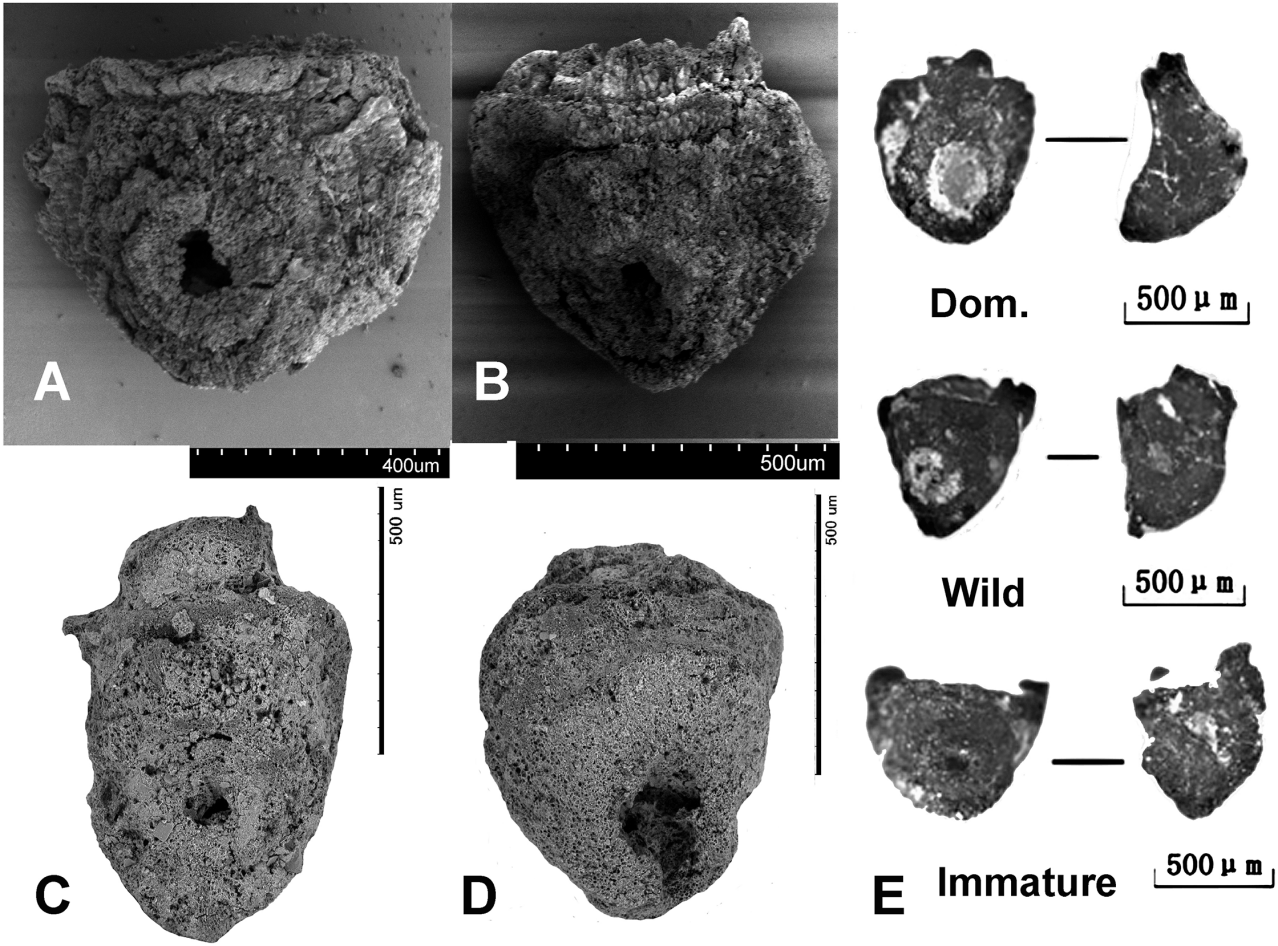
Examples of rice spikelet bases: (A) SEM image of domesticated type from H1601 (Longshan), (B) SEM image of domesticated type from H1601, (C) ESEM image of wild-type from H2000 (Pre-Yangshao), (D) ESEM image of domesticated-type from H2000, (E) photographed examples of domestic, wild and immature spikelet bases from H2000 (Pre-Yangshao).

Rice was examined further on the basis of spikelet bases and grain metrics to determine domestication status. Spikelet bases (Fig 8) were classified into domesticated type, wild type and immature type [30–32], of which 778 could be assigned, while 107 could not be assigned due to poor preservation condition. Criteria were developed through comparative study of 140 modern populations, domesticated and wild drawn from across China, Japan, Southeast Asia and India [30]. These criteria can be coherently related to the genetic basis of non-shattering adaptations in rice [31] These criteria have now been employed on numerous assemblages from India, China and Thailand producing a coherent picture of the long-term evolutionary trend of domestication [28; 32; 33]. Wild rice spikelet bases are characterized by a round, flat smooth scar, with a small round pore in the centre (Fig 8C and 8E); from the lateral view this surface is quite straight and flat. In domesticated rice the profile is usually uneven, the pore is much larger and uneven due the abscission area being wholly or partly torn out. The scar shape is often ovoid, wider than it is tall, rather than being round (Fig 8A, 8B, 8D and 8E). Immature spikelet bases are broken through the rachilla and retain a stub of attached rachilla or protruding vascular strands from the centre of the rachilla (Fig 8E). As noted in [30: supplementary information; 31] some broken rachillae like the immature type can be found in domesticated rice but a survey of 57 landraces found this trait in 33% of population and within those population accounted for only upto 5% of spikelet base, we therefor conclude that significant presence of such spikelets bases in early rice is likely to represent present of immature (green-harvested) spikelets. In addition, archaeological data indicate this morphotype declines alongside wild forms [30] which suggests that its presence in early sites is due to the inclusion of immature spikelets.

**Fig 9:**
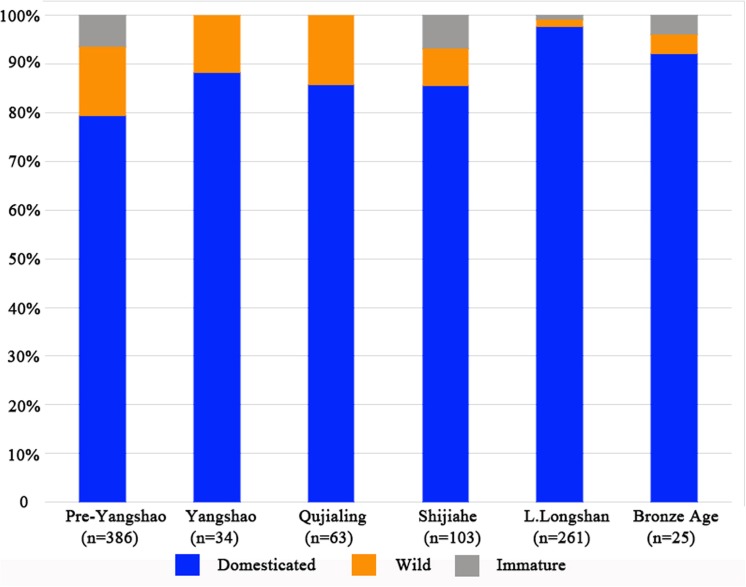
Proportions of identifiable spikelet base types in the phases of Baligang.

The proportion of domesticated type in all periods is over 80%, which means the earliest rice appeared at this site had already been domesticated (Fig 9). As a result, rice domestication in this region should probably have occurred before 8,300 years ago. However, one word of caution is warranted, as the domesticated spikelet bases themselves have not been directly dated, and some intrusiveness from overlying Yangshao levels cannot be ruled out entirely, but directly dated rice grains are consistent. Therefore the use of rice in the 7^th^ Millennium BC (9^th^ Millennium BP) is clear and is associated with at least some, if not mostly, non-shattering spikelets.

**Fig 10:**
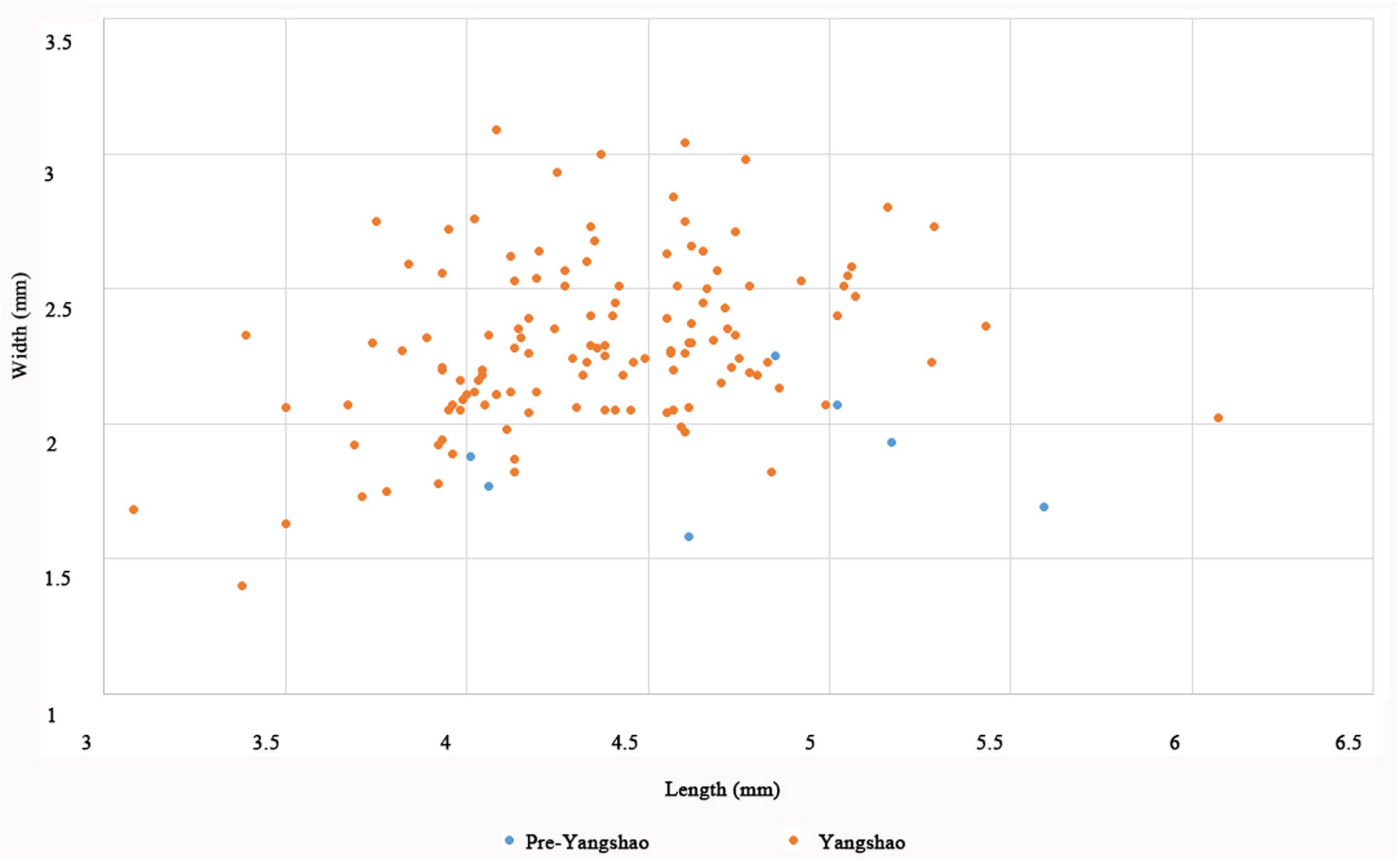
Scatter plot of length and width of measured rice grains from Pre-Yangshao and Yangshao period of Baligang site *(7 from Pre-Yangshao period*, *129 from Yangshao period)*.

**Fig 11:**
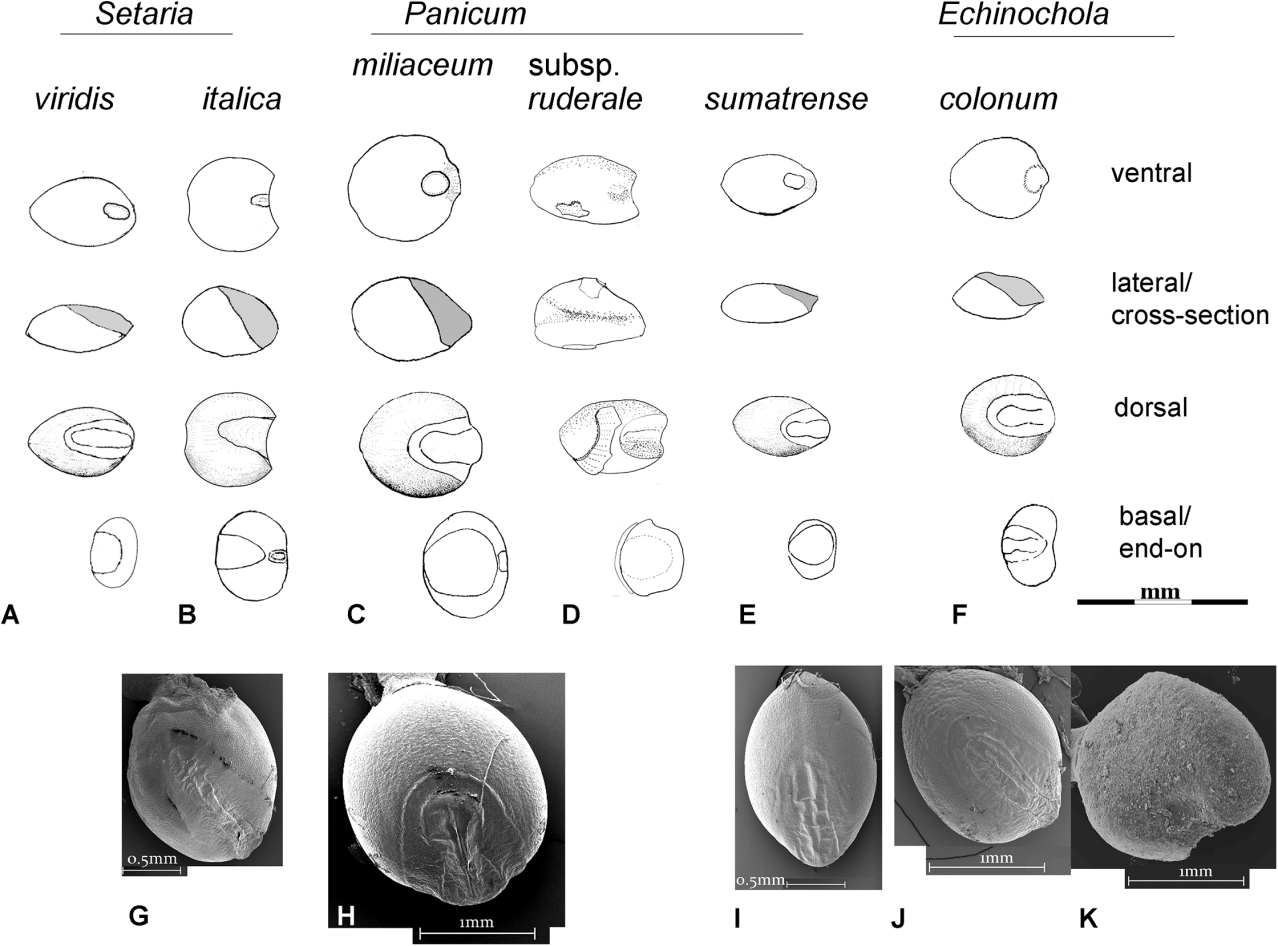
Rice grain metrics at Baligang: average value of length and width, and mean standard deviation indicated for each period.

**Fig 12:**
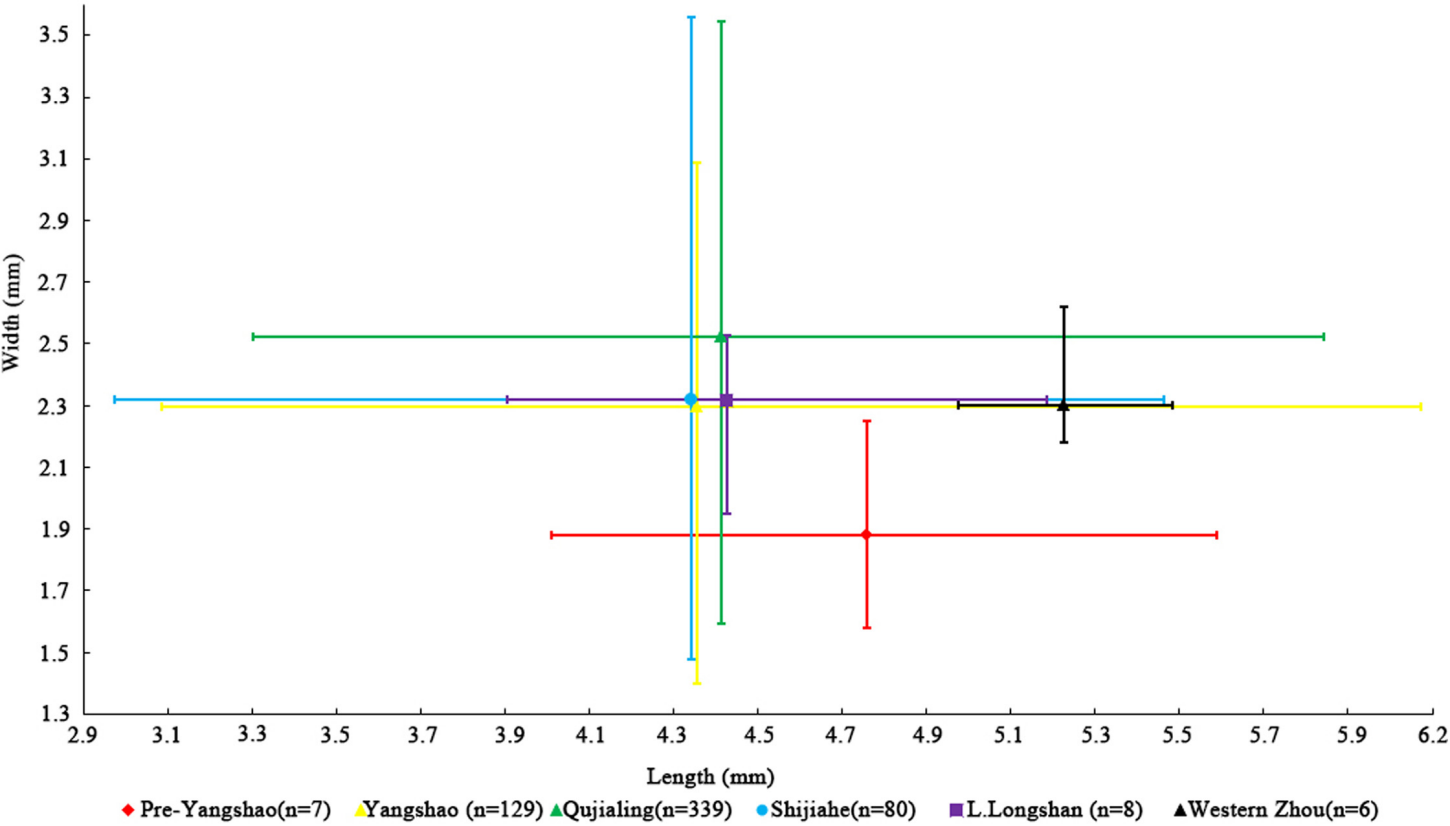
Comparison of L/W ratios of rice grains from different periods of Baligang site.

In addition, we measured 569 mature rice grains from different periods of Baligang site. Previous studies on rice grain morphology revealed the significant overlap between modern wild and domesticated rice populations, especially in grain length and length: width ratios [34]. Grain shape variation is also heavily influenced by local environmental factors and shrinkage due to carbonization. Thus on its own, this provides a weak indicator of domestication status [35, 36]. Nevertheless, time series data suggest directional change on population averages and ranges during the domestication episode, as for example documented in the Lower Yangtze between 6000 BC and 3000 BC [28, 30]. In the case of Baligang, it appears that Pre-Yangshao rice had not yet completed undergoing grain size evolution, as grains are thinner (Figs 10, 11 and 12). The high L:W ration of rice in the Pre-Yangshao was much more similar to that recorded in wild rice, whereas those in the Yangshao and later periods fit best with domestication *japonica* subspecies [33]. Intriguingly, the Bronze Age rice grains have a ratio somewhat closer to typical *indica* subspecies [33], which could indicate the introduction of a new variety or diversification within Chinese rice varieties.

**Fig 13:**
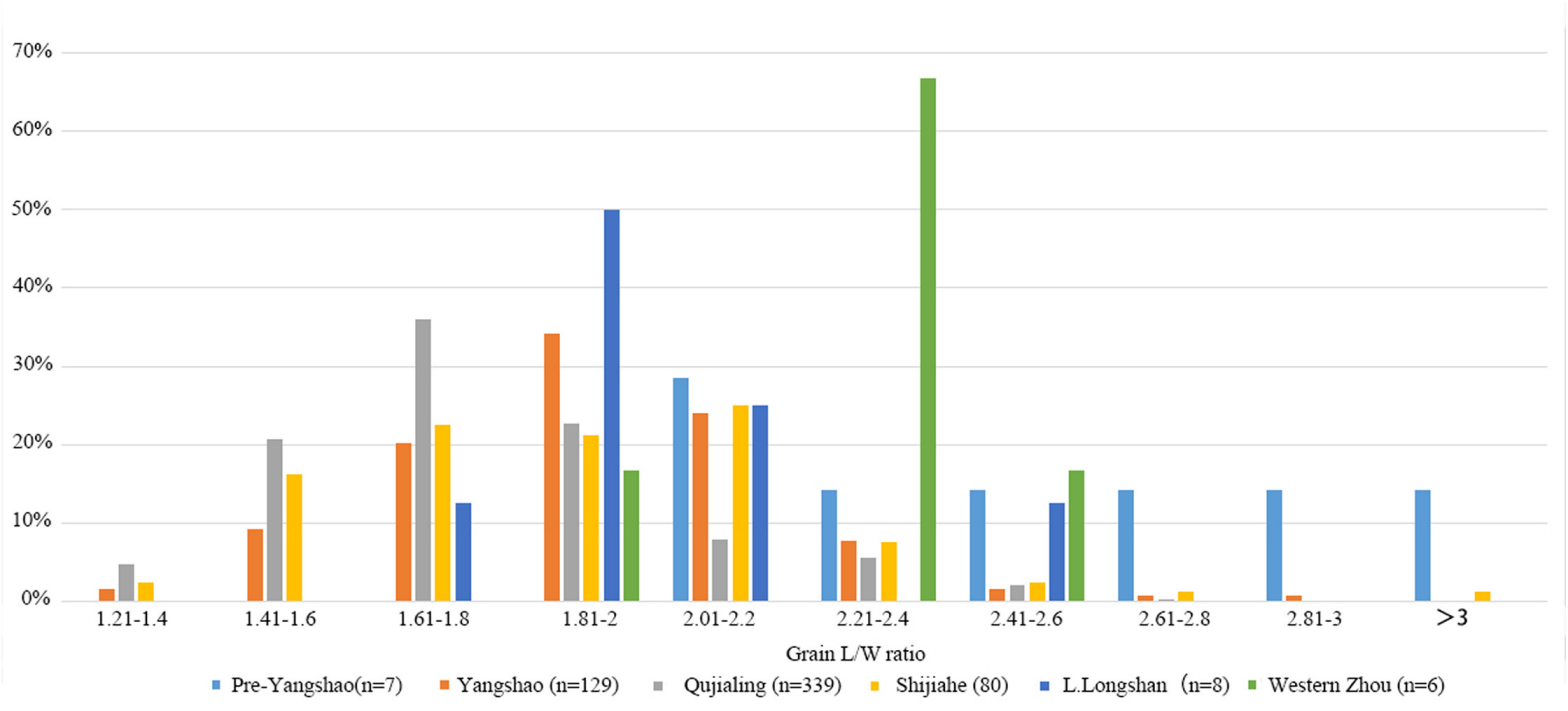
Drawings and scanning electron micrographs (SEMs) of representative reference material for millets illustrating identification criteria. Drawings arranged from top to bottom in central, lateral (longitudinal cross section), dorsal, and basal views, at the same scale: A. *Setaria viridis*, B. *Setaria italica*, C. *Panicum miliaceum*, D. *Panicum miliaceum* subsp, *ruderale* (drawn from archaeological specimen from Longshan period Xiawu, after ref [9]), E. *Panicum sumatrense* (Indian little millet, representative of small-grained *Panicum* spp., F. *Echinochloa colonum;* SEMs at the same scale, G. *Setaria italica* dorsal, H. *Panicum miliceum* dorsal, I. *Panicum sumatrense* dorsal, J. *Echinochlos colonum* dorsal, K. *Echionochloa colonum* ventral (note: wide hilum). All drawings and SEMs by DQF, selected as typical from amongst several populations of each taxon in modern reference material in the UCL archaeobotany reference collection (with the exception of the archaeological specimen in D).

Millet identification is often a challenge, especially when close wild relatives are likely to be present. In our current material we have separated wild type foxtail millets (*Setaria viridis*), some willd type *Panicum sp*. as well as barnyard grass (*Echinochloa* sp.) and immature (unfilled) grains of both *Setaria* and *Panicum*. Although separation of domesticated *Setaria italica* from *Panicum miliaceum* is well-established [e.g. 9, 37], the criteria used here also take into account the likely presence of wild/weedy forms of millets and immature grains. The criteria applied to separating these categories were outlined in [7] and are illustrated in Fig 13, with a detailed consideration of recognizing immature *S*. *italica* and *P*. *miliaceum* published in [38]. *Setaria* and *Panicum* are separated based on the length scutellum or embryo area [Fig 13], which is long in *Setaria* (around 65–70% of grain length) and shorter in *Panicum* (45–50%% of grain length). The *Panicum* scutellum is generally wider and rounder (Fig 13C, 13D, 13E, 13H and 13I). In mature crop grains *Panicum* is large with charred archaeological examples typically 1.5mm or more, while *Setaria* are from 1.1–1.5mm. Domesticated taxa tend to be rounded and wider than their wild relatives, and grain widening and thickening (rather than lengthening) seems to be a recurrent pattern with cereal domestication [28]. Both domesticated *Setaria* and *Panicum* have grain length: width ratios around 1:1 (Fig 13B and 13C), whereas wild relatives are much longer, e.g. ~1.35 in *S*. *viridis*; *~*1.5 in many *Panicum* spp. (Fig 13A, 13D and 13E). Wild taxa also have a more acute apex. Immature grains are notably flatter, but have similar scutellum size and shape, and only very immature examples are also thinner (elongate) [38]. Wild *Echinochloa* grains are superficially similar to *Setaria* in having a long scutellum, but they differ in having a maximum breadth displaced towards the basal end of the grain (towards the hilum) and being dorsal-ventrally compressed; they also have a hilum that is wider than it is long (Fig 13F, 13J and 13K). In the case *Setaria viridis*, the wild progenitor of foxtail millet, identifications are quite secure and modern reference material is readily available. In the case of *Panicum miliaceum* the wild ancestor remains uncertain [1]. A candidate is *Panicum miliaceum* subsp. *ruderale*, which occurs widely as a weed across Eurasia, and has been provisionally identified in material from northern Henan [9]. However, well-studied modern populations from eastern Europe show patterns of shattering that are adaptations to agriculture and suggest evolution of at least some *P*. *ruderale* populations from feralization of the crop [39]. It is unknown if morphotypes similar to this would have existed in the wild before the origins of cultivation. The wild *Panicum* sp. in the current material is smaller than the *P*. *ruderale* types in the Longshan period of the Ying valley [9] (compare Fig 13D and Fig 4I) and it is unclear whether these should be regarded as part of the wild progenitor complex of *P*. *miliaceum* or just some other wild *Panicum*.

### Fruits

Acorns (*Lithocarpus* sp., *Cyclobalanopsis* sp. or *Quercus* sp.), were the only tree nut from Baligang site (Fig 5M). Acorns were likely a very important food resource in the Pre-Yangshao period, as they have found to be in the archaeobotany of the Lower Yangtze and the Huai valleys [30, 34, 40]. Samples from H2000 (a midden-filled pit) contain large amounts of acorns, which take up more than 17% of all plant remains in the Pre-Yangshao period. However, in the following periods, except for one fragment found in one sample of Shijiahe period, acorns did not appear in any other samples. Endocarp fragments of tree fruits including peach (*Amygdalus persica*), apricot (cf. *Armeniaca vulgaris*), and persimmon type *Diospyros* cf. *lotus* (Fig 5l) appeared in very few samples with quite low frequencies. A similar situation of low ubiquity and frequency also can be observed in remains of *Cucumis melo* and *Rubus* (Fig 5k). Besides, small fragments of possible water chestnut (cf. *Trapa sp*.) were collected from two samples from the Yangshao period (Fig 5I and 5J), two samples from the Shijiahe period, one Late Longshan period sample, and one sample from the Western Zhou dynasty phase. Nevertheless, the frequencies were always quite low, which indicates aquatic wild resources were not important in daily food of people living at Baligang site; this is in contrast to the importance of aquatic nuts like *Trapa* and *Euryale* in the subsistence of the Lower Yangtze Neolithic [30, 34, 36].

### Field weeds

**Fig 14:**
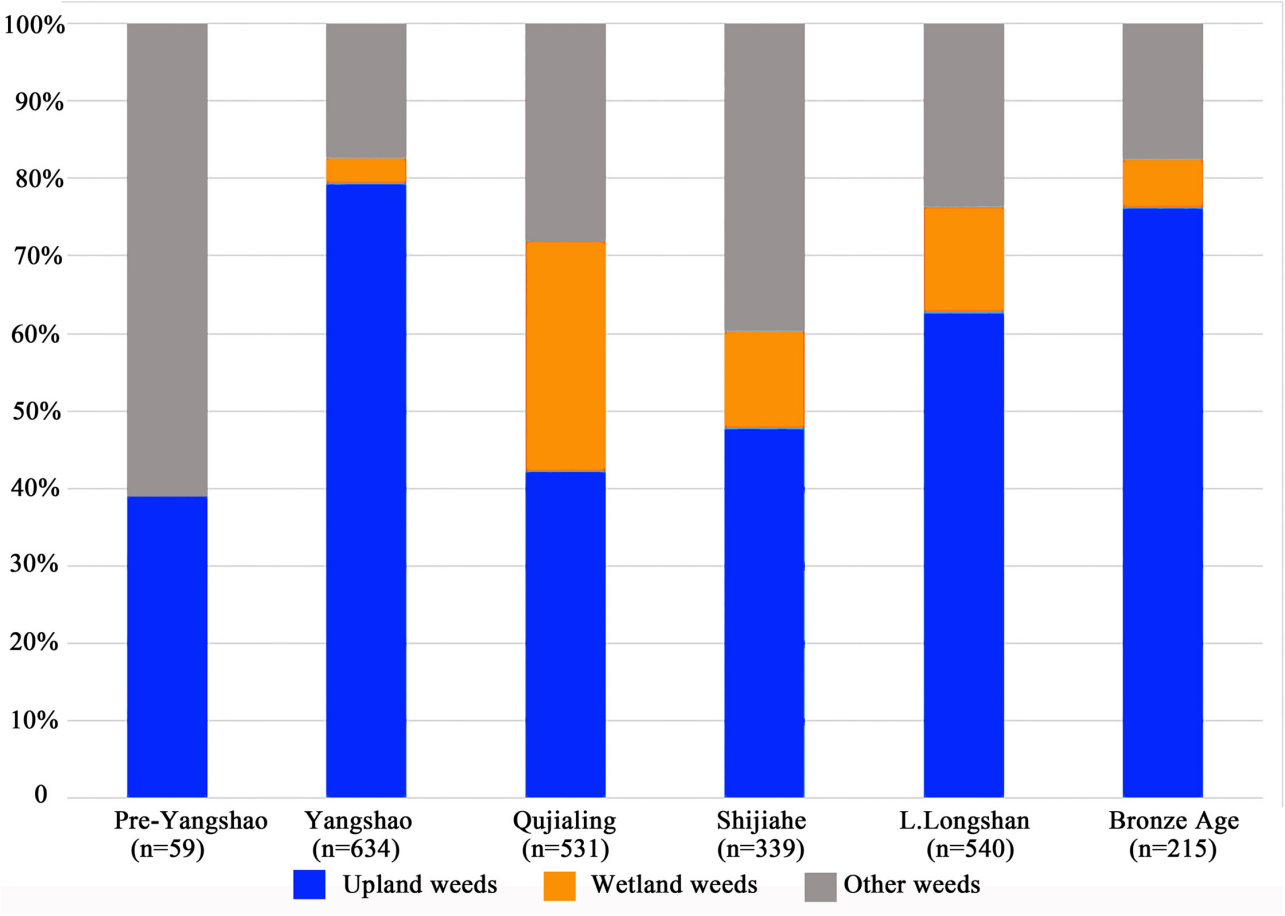
Proportion of different weeds in all periods of Baligang site (Extreme outlier samples excluded).

**Fig 15:**
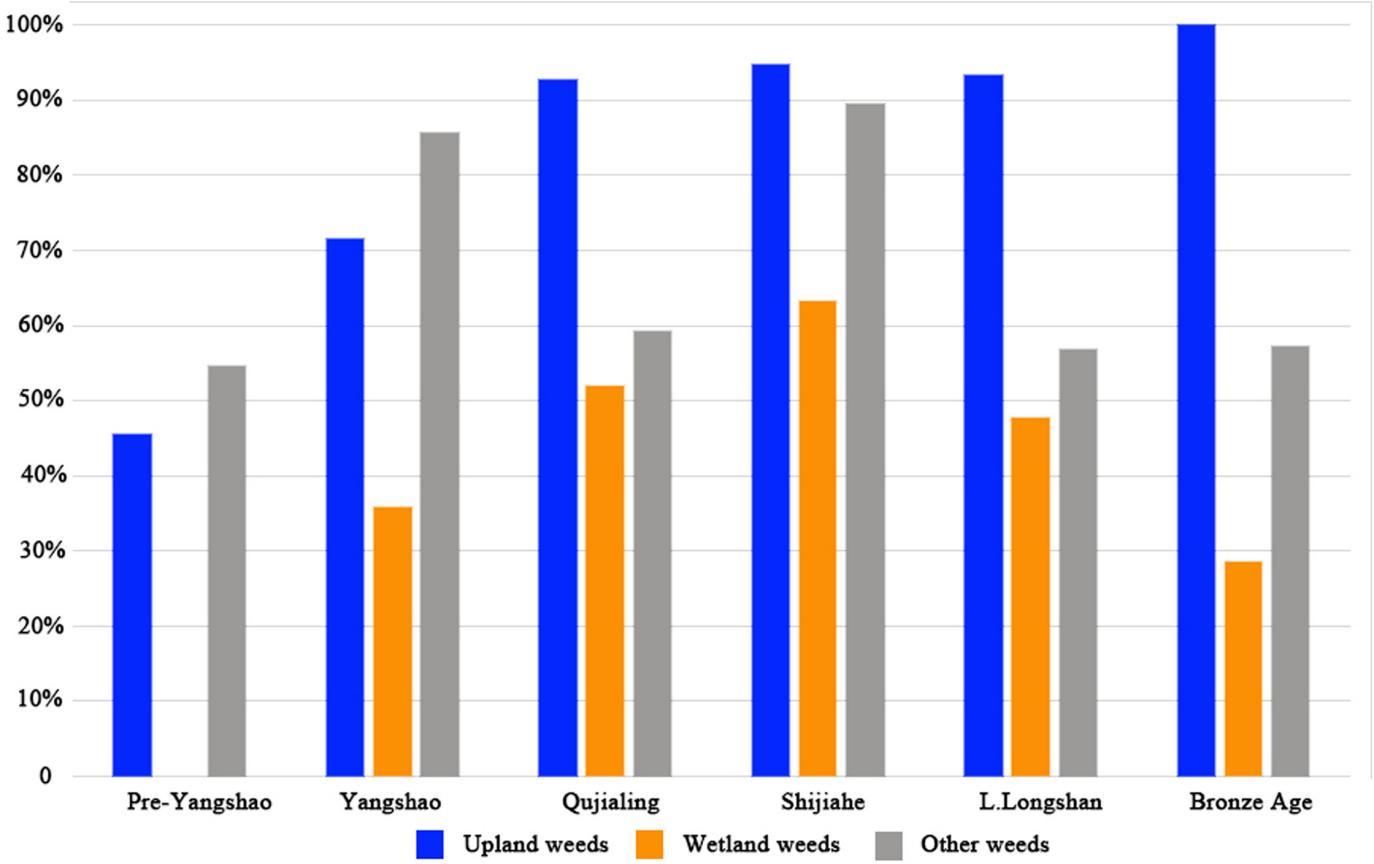
Ubiquity of different weeds in all periods of Baligang site.

5318 seeds of possible field weeds were collected from all Baligang samples, which occupied more than half of all the plant remains. Based on the present growing ecology of these species [41, 42], all identified weeds are classified into three groups, the wetland group, the upland group and other weeds group. The wetland group includes typical paddy field weeds and weeds growing in both paddy field and/or other wetland. While the upland group covers the typical upland field weeds and those growing both in the upland fields and other places. The rest are all classified into other weeds group, including wasteland weeds, roadside weeds and forest side weeds, all of which seldom appear in any type of farmland today. This highlights that weed flora has evolved over time and some taxa that were likely weeds in prehistory are no longer common on arable land. Overall, the species of wetland group are quite limited, just *Echinochloa* sp. and different genera of Cyperaceae. Several of these taxa are tolerant of damp or periodically wet ground, and sedges are known to range into the drier forms of rice cultivation (i.e. upland rice) [43]. However, the upland group includes 8 kinds of grasses as well as 10 kinds of weeds in other families. Thus the upland weeds are much more diverse. The proportion of upland weeds was also much higher than wetland weeds in all periods (Fig 14), and upland weeds also have higher ubiquity (Fig 15). Modern analogue studies of weed flora in different rice ecologies found that weed diversity was highest in direct cultivation systems, with high diversity especially amongst dicotyledonous weeds and sometimes grasses [43]. The strongest signal of wetland weeds comes from the Qujialing followed by the Shijiahe periods. In contrast to the Yangshao period this may in part be due to the increase of rice relative to millets. Nevertheless the high diversity of upland (dry) weeds in the Pre-Yangshao and throughout suggest that at least some, if not much, of the rice was grown under somewhat drier conditions. We expect such conditions under rainfed cultivation or irregularly flooded parts of the flood plain, including the drier end of decrue systems, i.e. those systems sown during or after the recession of seasonal floodwaters. Low intensity management of flood plain rice cultivation can be expected to include both some wetter and some drier zones within the same cultivation systems [43]. It should also be noted that cultivation in drier environments than the wild progenitor is postulated to have promoted increased grain yield under early cultivated rice, suggested for example by Late Majiabang era paddy fields in the Lower Yangtze [6, 8]. The drier ecology represented by the Pre-Yangshao samples at Baligang may also indicate this phase of drier cultivation that was necessary for the evolution of domesticated *japonica* rice.

## Discussion

### Rice domestication: new evidence on the Middle Yangtze pathway

Rice has long been believed to have been domesticated in the middle and lower Yangtze valley [3, 14, 40, 44–47]. With the improvements of methodology and accumulation of new archaeobotanical evidence, a multi-centric model of rice origins in China becomes more likely [8]. Apart from the middle and lower Yangtze valley, the Huai River valley, and the Houli culture of Shandong also emerged as possible cultivation centers of early rice. Recent systematic archaeobotanical research conducted in the Lower Yangtze region revealed rice domestication is a very long process, which probably started 10,000–8,000 years ago, and eventually finished around 4000 BC with the fixing of domestication traits [28,30,36]. However, except for the Lower Yangtze valley, the developing progress in other regions still remains unclear, due to a lack of hard evidence.

In the Huai River valley, Jiahu is the only site with evidence of early rice remains, as well as apparent stone spades for cultivation [19, 40]. More than 400 rice grains were collected from systematic flotation work, but no rice spikelet base were found in any sample [1, 40]. Therefore domestication status in terms of non-shattering has remained unclear. On the other hand, the grain shape of Jiahu rice was highly variable, and its wild or domesticated affiliation has been debated [34, 36, 40, 48–51], and no clear trend in the change of rice grain shape from the early phase to the late phase of this site. As a result, it is difficult to evaluate the status of the Huai River valley in the domestication process of rice, although it is likely early pre-domestication cultivation was practiced here [51]. Rice grains belonging to the Houli culture of Shandong, have been found in small numbers from Yuezhuang and Xihe. Research on plant and animal remains of these two sites reveal this period included exploitation of millets and rice, as part of an economy that included fishing, hunting and gathering [52–54]. The measurements of the only 9 whole rice grains of Yuezhuang site, indicates that they are small and more in keeping with a wild type [53]. Furthermore, there is no clear evidence of rice cultivation has been found in the subsequent Beixin and early Dawenkou culture periods of this region [8, 36, 55]. Therefore, rice grains from Houli culture of Shandong should be treated as evidence of wild rice utilization, and they do not appear to relate to any later tradition of rice cultivation.

The middle Yangtze valley is considered as a key region of rice domestication. Some cave sites have evidence for rice exploitation back to almost the Last Glacial Maximum, 16,000–18,000 years ago, from cave sites with phytoliths such as Xianrendong, Diaotonghuan and Yuchanyan [56–60], but these may bear no relationship to early cultivars or the domestication process evidence in Holocene sites [51, 59–63]. Large amounts of rice husks and grains were collected in the middle Neolithic sites like Pengtoushan and Bashidang site, dating between 8000 and 6000 BC [51, 63, 64], but at present, no data of systematic flotation or spikelet base morphology is available from these sites. Grain metrics of these sites fit better with morphologically wild rice [33, 34, 36]. Nevertheless, systematic research of plant remains of the Daxi period (ca. 4500–3500 BC.) at the Chengtoushan site in this region shows rice cultivation was a very important subsistence strategy in that period, including wet rice weed flora [13], and grain metrics that fit with domesticated rice, or rice undergoing domestication [34, 64–66]. At the same time, foxtail millet had spread into this region, possibly cultivated as a risk-buffering crop in different ecological areas from rice [13]. Interestingly, the paddy fields of Pre-Daxi period (5000–4500 BC) Chengtoushan show the area of each paddy field is larger than that found in the same period of the Lower Yangtze valley [6, 36], which points to more advanced rice cultivation technology in the Middle Yangtze region, and a different tradition of field systems than in the Lower Yangtze region. All these clues remind us that the middle Yangtze region is potentially another important area of rice domestication, and the evolutionary process there may have begun earlier than in the Lower Yangtze valley [8]. Baligang, located on northern periphery of the middle Yangtze region has provided new evidence that domestication, in terms of non-shattering, may have occurred in this region about two millennia before a parallel process in the Lower Yangtze.

Rice domestication in Nanyang appears to represent a distinct trajectory from that of the Lower Yangtze. While we lack evidence for a trajectory of directional change in domesticated spikelet base frequency, selection for non-shattering would appear to have already been completed by the middle of the 7^th^ millennium BC, and certainly by 6300 BC. By contrast, in the Lower Yangtze valley, where the proportion of domesticated rice spikelet base increased from 27.4% to 38.8% over 300 years at Tianluoshan site from 4900 BC to 4600 BC [30], and was even lower at Kuahuqiao [28], and had levelled off at >70% by ca. 4000 BC. After this period, the proportion of domesticated types maintains more than 80% at many sites in the lower Yangtze valley [28, 67].

Domestication related change in rice grain size, however, was not yet completed in the Pre-Yangshao period at Baligang. The measurements from Baligang are narrower, indicating that further evolution in domestication characters took place after the Pre-Yangshao period, although it is not preserved within the stratigraphy of the Baligang excavations. Grain width in early phase at Baligang is smaller than at Kuahuqiao and significantly smaller than Tianluoshan or later sites in the Lower Yangtze [68]. However, given the 2,000 year hiatus between the Pre-Yangshao and Yangshao period, the rice from the Yangshao and later could represent a strain introduced from elsewhere rather than *in situ* evolution of grain size.

**Fig 16:**
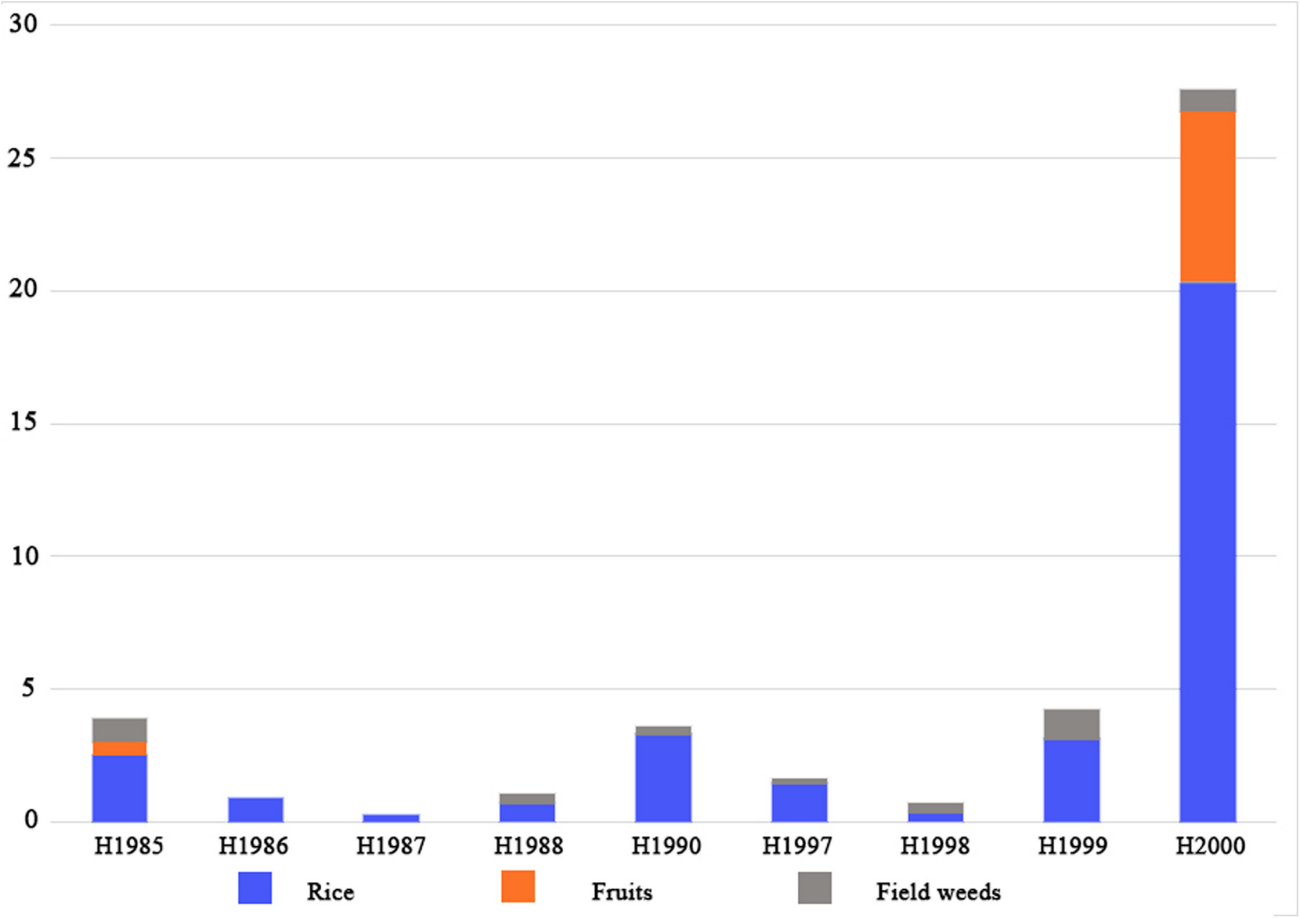
Density of plant remains of Pre-Yangshao period at Baligang site (count per 10 liters, outliers excluded). 1 cf. immature broomcorn millet grain and 2 wild soybean were found in H2000. In H1992, the densities of rice remains and field weeds are 63 per 10 liters and 13 per 10Liters respectively.

Despite the fact that domesticated rice was consumed at Baligang in the Pre-Yangshao period, the subsistence strategy still included an important wild food gathering component. Rice was widely discovered in nearly every context of the Pre-Yangshao period, but the density in most contexts is actually quite low (Fig 16), and over 67% of the remains are rice spikelet bases. On the contrary, acorns were found in large quantities from context H2000, mainly as charred fragments of nutmeat. Therefore the rice is represented by processing waste, while the nuts are represented by edible product that has been accidentally charred. Besides rice and acorns, other edible plant resources are just one broomcorn millet grain (immature or plausibly wild) and two fragments of wild soybean. Therefore, plant remains from the Pre-Yangshao period of Baligang generally demonstrate a mixed subsistence strategy based on rice and acorns. The economy was therefore transitional, with an important component of gathered nuts (acorns) as found in other regions, including Jiahu site [40, 51], Pengtoushan and Bashidang site in the middle Yangtze valley [63] and Kuahuqiao site in the lower Yangtze valley [41, 62, 66, 69–71], and even sites of Peiligang culture in north China [72–73]. All of these culture included wild fruits and nuts, especially acorns. Acorns disappear from later, more clearly agricultural periods.

In the Yangshao and the following periods, People living at Baligang focused only on multi-crop agriculture for their staple foods, including millets from the north and rice. Due to the ~2000 year hiatus in the Baligang sequence, we are unable to determine when wild starchy foods were given up for a full commitment to cereal agriculture. To the south in the middle Yangtze valley, on the Liyang Plain, a similar contrast can be found between the Pengtoushan period (7000–6000 BC) and the later Daxi (4500–4000 BC). The gathering of wild foods, like acorns, water chestnuts, *Euryale ferox* and so on, is evident at Pengtoushan period Bashidang [65], like at Baligang. However, in the Daxi period at Chengtoushan, if we examine the species of fruits they exploited, no fruits rich in starch were discovered, while berries especially *Rubus* are the most important, which can only be used as subsidiary food rather than a staple [13, 29]; the staples were rice and foxtail millet, like at Baligang, but without evidence for any *Panicum miliaceum*. Therefore, reliance on cultivated domesticates, which we can regard as full agriculture, is evident at Daxi period Chengtoushan and contemporary Early Yangshao Baligang.

We therefore conclude that although the transition to an agricultural economy may have taken place at around the same time in the middle and Lower Yangtze, ca. 6000 BP, but the preceding trajectory of domestication differed. In the Lower Yangtze, full agricultural dependence is attributed to the Late Majiabang, with the appearance of field systems and the decline in starchy nuts. What appears different is that morphological domestication was fixed during this shift to agriculture in Lower Yangtze. By contrast at Baligang, the Pre-Yangshao evidence would imply a lag time for ~2000 years between the domestication process, selection for non-shattering, and the shift to agricultural reliance.

### Crop choices in relation to cultural geography

After the establishment of the exclusive status of farming in the subsistence of Yangshao period, there were still changes within the farming practices that can be observed from the plant remains. In the Yangshao period, rice, foxtail millet and broomcorn millet were the three main crops cultivated. Analysis of proportions and ubiquities of these crops shows they were nearly equally important in the crop assemblage (Figs 6 and 7). This mixed farming strategy was continuously practiced, but two distinct changes can be observed. One happened between Yangshao and Qujialing period, and the other in the late Longshan period. The first change was from a totally mixed agriculture pattern to a rice-focused pattern, with the decline of millets in the Qujialing and Shijiahe periods. This seems to reflect the material culture affiliation with the middle Yangtze to the south, with an emphasis on southern rice cultivation. In the late Longshan period, when a northern material culture association is once again evident, millet increased, reflected by a slight increase in relative frequency, but a marked increase in ubiquity. This trend continued after the Longshan period and finally ended with the formation of a foxtail millet based agriculture pattern in the Bronze Age, which also can be suggested to have had a stronger cultural influence from the Yellow River region of the Zhou state. Changes in phytolith assemblages at Baligang also suggest shifts in the importance of millets and rice associated weed flora that suggest the Yangshao and Late Longshan phases were more similar to Yellow River archaeobotanical assemblages than the Yangtze-influenced Qujialing and Shijiahe assemblages [25]. So we see a clear correlation between archaeology culture changes and crop choices at Baligang site, and the change of crop proportions seems to best reflect the cultural preferences of different regional traditions influencing the inhabitants of Baligang.

### Arable weeds and crop habitat conditions

As a preliminary form of analysis, we divided all field weeds, or plausible field weeds, into three groups, the wetland group, the upland group and other on the basis of descriptions in modern Chinese agricultural treatments [41, 42]. As can be seen in Figs 14 and 15, obligate wetland weeds are missing from the Pre-Yangshao period. While it is possible that some of these seeds are not actually from weeds, given the inference that wild food collecting was still prominent, we infer that rice was grown under relatively drier condition, such as through opportunistic cultivation of the marginal and drier parts of the flood plain, and even though raindfed cultivation, which would have been facilitated in this period by wetter climatic conditions in the 7^th^ millennium BC [62, 74–77]. Annual rainfalls in the past 20 years of Nanyang basin were between 558 to 1263mm, and in most years it was actually lower than 800mm, which is the minimum level for non-flood rice cultivation [78]. From the reconstruction of precipitation pattern in the Holocene, we know that the annual rainfalls were 250–300mm higher than today in the middle and lower Yangtze River throughout the Holocene Megathermal (ca. 5,400–4,300 BC.) [79]. Therefore it seems likely that after the Yangshao period rice could have only been grown successfully under well watered and even irrigated conditions. The rise in wetland weeds from the Yangshao through the Shijihe period would seem to support this conclusion with a likely start of wet cultivation systems in the Yangshao period. The high levels of dryland weeds are likely to reflect millet cultivation, in addition to the drier margins of rice cultivation. Phytolith analyses also indicate wet rice cultivation from the Yangshao, with more intensive and better irrigated rice inferred for the Qiujialing and Shijiahe periods [25].

## Conclusion

Plant remains from Baligang site provide us with full details of subsistence strategy in Neolithic period and Bronze Age as well. Rice had been domesticated before 6300 BC according to the morphological characteristics of rice spikelet bases, although evolution of grain shape was not yet complete, and the transition to an agricultural economy with reduced reliance of wild starch rich nuts had not yet occurred. This was not full-fledge farming in economic terms but it was cultivation of a domesticated form of rice. After a hiatus of around 2000 years, the site was re-occupied by sedentary agriculturalists of the Yangshao tradition who practiced mixed farming of foxtail millet and broomcorn millet. As a response to cultural changes, crop assemblages varied in different periods along with the interaction between north and south China, with more millets grown in period under the cultural influence of the north. Furthermore, given the relatively dry climatic conditions of the Nanyang basin, wetland weeds recovered from the site are interpreted as coming from rice paddy fields, with wet rice cultivation starting in the Yangshao and intensifying through the Qujialing and Shijiahe periods. Rice cultivation was greatly reduced in the Late Longshan period which saw the reintroduction of cultural influence from the north, the rise in importance of the millets and the introduction of wheat.

Baligang provides a long sequence that registers many of the key trends in the Neolithic agriculture of central China. This includes evidence of rice cultivation alongside wild acorn consumption in the 7^th^ millennium BC, even if the new evidence suggests that morphological domestication was more advanced at Baligang than in the Lower Yangtze at that time. In the Yangshao millets were important to sedentary agriculture and mixed farming that included rice and millets becomes well established for the first time, although rice was much less prominent in more northerly sites [9–11]**10**. Intensive wet rice cultivation can be associated with Middle Yangtze cultures of Qujiling and Shijiahe, period that saw the expansion of human population and social complexity in the middle Yangtze region. Soybeans, presumably domesticated in the Yellow River region [27, 28] were adopted in Shijiahe time from the middle of the third millennium BC. Wheat arrived in small quantities around the start of the Second Millennium BC [42–44], along with re-emphasis on millets, associated with the Longshan culture. Baligang therefore provides an important archaeobotany type site for comparisons with the better-studied Yellow River and the middle Yangtze, and highlights links between them such as the spread of soybean and wheat. However, more comparable data are needed from the South in order to understand Middle Yangtze subsistence. The tantalizing new evidence for domestication rice at Baligang site before 6300 BC require data from other sites and periods to be put into an evolutionary trajectory from rice gathering to domestication.

## Supporting Information

S1 Table The raw archaeobotanical data from Baligang, with individiula flotation samples in columns and counts of items by taxon in rows. Taxa names/categories are listed in column D. Samples are grouped by phase, numbered according to onsite context numbers, and sample size (approximate volume of sediment floated) is indicated. The first three columns indicate how we have classified probable arable weeds (wetland, upland, or other).
(XLSX)
